# Hydroxypropyl Methylcellulose as a Mucoadhesive Polymer in Ethanol-Free Buprenorphine Gel for Neonatal Sublingual Delivery

**DOI:** 10.3390/polym18040435

**Published:** 2026-02-09

**Authors:** Sanskruti Dave, Viren Soni, Samarth A. Shah, Walter K. Kraft, Gagan Kaushal

**Affiliations:** 1 Department of Pharmaceutical Sciences, Jefferson College of Pharmacy, Thomas Jefferson University, Philadelphia, PA 19107, USAvsoni08034@gmail.com (V.S.);; 2 Department of Pharmacology, Physiology & Cancer Biology, Sidney Medical College, Thomas Jefferson University, Philadelphia, PA 19107, USA

**Keywords:** buprenorphine, drug stability, ethanol-free sublingual gel formulation, neonatal opioid withdrawal syndrome, penetration enhancement

## Abstract

Buprenorphine (BUP) is widely used in the treatment of neonatal opioid withdrawal syndrome (NOWS). However, the most compounded formulation contains 30% ethanol, despite regulatory and clinical concerns regarding ethanol exposure in pediatric patients. Thus, this research aimed to develop an ethanol-free sublingual (SL) gel formulation of BUP that would be safe, stable, and suitable for NOWS. Multiple polymers were screened as gelling agents, with hydroxypropyl methylcellulose (HPMC) emerging as the ideal base polymer for the formulation due to its optimal pH, rheological characteristics, and stability. The formulated gels were stored at room temperature and refrigerated conditions for 30 days and evaluated for stability using pH, rheology, and liquid chromatography-mass spectrometry. BUP content was between 90–110% of the labeled amount of the dosage form (75 µg/mL) at all time-points, and the pH remained close to physiological values. Release studies demonstrated a drug release of 23–24% for SL gels without surfactants stored at room temperature and refrigerated conditions, respectively. Incorporation of non-ionic surfactants (Tween 20 and Tween 80) significantly increased drug release to 33% and 40%, respectively, reflecting enhanced solubilization and improved mucosal penetration. The ethanol-free formulation demonstrated physicochemical stability and favorable release characteristics suitable for neonatal administration. These findings represent a meaningful advance in the development of safer pediatric formulations for NOWS.

## 1. Introduction

The incidence of neonatal opioid withdrawal syndrome (NOWS) has continued to rise since the early 21st century due to increased prenatal opioid exposure. Affected neonates exhibit symptoms such as irritability, poor consoling, and difficulty feeding. Initial management typically includes non-pharmacological measures, such as swaddling, a low stimulus environment, and maternal involvement. However, when symptoms interfere with feeding, weight gain, or bonding, pharmacological intervention becomes necessary [1].

Buprenorphine (BUP) [2], a Biopharmaceutics Classification System (BCS) Class III drug, has distinctive pharmacology, demonstrating affinity for both µ and κ opiate receptors. BUP is considered safer than other opioids like methadone because it has a ‘ceiling effect’—meaning its pain-relieving effects level off at higher doses, which helps reduce the risk of overdose [3].

The most widely used formulation for treating NOWS is 75 µg/mL of BUP in 30% ethanol compounded from BUP injection (0.3 mg/mL) and administered sublingually [4]. Our group’s previous work involved compounding ethanol-free BUP oral syringes and establishing BUP’s stability via physicochemical testing for 60 days. This formulation has been used in clinical care in an optimized NOWS study and was incorporated into part of standard care [5].

BUP undergoes significant first-pass metabolism, which lowers its bioavailability and necessitates frequent administration. The sublingual (SL) venous drainage can serve as an advantage, as it enhances the bioavailability of the drug substance being used. The SL permeation mechanism involves two primary pathways: (1) the lipoidal intercellular route, which allows for the transport between cells through polar lipids; (2) the aqueous transcellular route, which allows for transport through the cells. The aqueous channel is a paracellular conduit; however, water molecules are trapped by the polar heads of the intercellular lipids between cells [6].

Based on their described mechanisms of permeation for drug delivery, SL formulations allow for precise dosing of the drug substance and flexible dose regimens, and they may be tailored to the patient’s requirements. Additionally, gel formulation provides an avenue in terms of easier administration compared to other formulations. Using this dosage formulation would increase the absorption through the oral mucosa and the bioavailability of the active pharmaceutical ingredient, making this a suitable approach for treating NOWS.

Thus, we pursued an ethanol-free SL gel formulation of BUP with the incorporation of permeation enhancers and surfactants such as polysorbates. In this study, the goal was to develop an ethanol-free SL gel of BUP suitable for administration to neonates that would improve BUP release without compromising safety or physical stability. The stability of BUP in the developed dosage form was evaluated via physicochemical testing, along with drug release kinetics and functional group analysis of the formulation for 30 days.

## 2. Materials and Methods

### 2.1. Polymer Selection and Preparation of Placebo Gels

Different biodegradable polymers were evaluated for the desired SL gel formulation. A placebo gel was prepared for each polymer, and the polymers considered for the formulation were carbomer, hydroxyethyl cellulose (HEC) 250, and hydroxypropyl methylcellulose (HPMC). Placebo gels of 2% *w*/*v* of each polymer were formulated and screened to determine which polymer would be most suitable for the desired SL gel formulation.

### 2.2. Preparation of Placebo Carbomer Gels

The respective polymer was added to deionized water in a beaker slowly to avoid any clumps. The solution was placed on a hot plate with a magnetic stirrer at 40 °C until a homogenous solution was obtained. The solution was allowed to reach room temperature and left overnight to complete the gelling process.

### 2.3. Preparation of Placebo HEC 250 H and 250 M Gels

HEC 250 H and HEC 250 M were slowly added to deionized water in separate beakers, avoiding any clumps. The solutions were placed on a hot plate with a magnetic stirrer at 70 °C until a homogenous solution was achieved. Once homogenized, the solutions were allowed to cool to room temperature overnight, and a gel was obtained.

### 2.4. Preparation of Placebo HPMC Gels

HPMC was added slowly to deionized water in a beaker, avoiding any clumps. The solution was placed on a hot plate with a magnetic stirrer until a homogenous solution was reached. The resulting homogenized solution was kept overnight to allow for the formation of the desired gel.

### 2.5. Preparation of Placebo HPMC-Polysorbate Gels

To prepare the improved gel formulation, 2% *w*/*v* of HPMC was weighed and dissolved in deionized water in separate beakers. Additionally, 0.1% *w*/*v* of Tween 20 and Tween 80 were added to separate solutions slowly, along with the base polymer. Continuous stirring using a magnetic stirrer on a hot plate was performed, and the homogenized solutions were kept overnight to ensure complete gel formation.

### 2.6. Preparation of Drug-Loaded HPMC Gels

An SL gel of BUP was prepared using commercially available Buprenex^®^, 300 µg/mL (Lot no. 67737) from Par Pharmaceutical (Chestnut Ridge, NY, USA). For each formulation, 8 mL of Buprenex^®^ was transferred into a beaker (Solution A) and diluted 1:4 with water for irrigation to attain the desired drug concentration (75 µg/mL). Water for irrigation (24 mL) was measured and transferred into a separate beaker(s) (Solution B). For HPMC-only gels, HPMC was weighed to achieve a 2% *w*/*v* concentration and added to solution B. For HPMC-polysorbate gels, HPMC and polysorbates were weighed to achieve final concentrations of 2% *w*/*v* and 0.1% *w*/*v*, respectively, and added to solution B. Solutions A and B were mixed thoroughly, and the resulting solution was stirred until homogeneous. All formulations were left overnight to allow for the formation of the BUP-loaded SL gels.

### 2.7. Stability Study Design

The prepared SL gels of BUP were refrigerated (2–6 °C) or stored at room temperature (25 °C, 60% relative humidity). Three gels from each storage condition were assessed for physicochemical stability by measuring pH, viscosity, drug concentration, and drug release on days 0, 7, 15, and 30. The formulated gels were protected from light exposure, and the storage temperatures and humidity conditions were closely monitored throughout the study.

#### 2.7.1. pH Evaluation

Three SL gels of BUP at each storage condition were assessed for pH changes at each sampling time point using a Mettler Toledo Inlab^®^ Ultra-Microelectrode pH meter (Mettler Toledo Exact Equipment; Washington Crossing, PA, USA). The instrument was calibrated using a three-point standardization, with 3 buffer solutions (pH of 4.0, 7.0, and 10.0) as per USP <791> guidelines [7] over 30 days.

#### 2.7.2. Rheological Characterization

The viscosity of the gel formulation was measured using an AR-G2 Rheometer (TA Instruments, Wilmington, DE, USA). The geometry was selected based on the type and quality of the gel. Geometry, rotational mapping, and gap calibrations were performed before use. The instrument was calibrated using S600 silicone oil (Cannon Instrument Company, Lot no: 19201; State College, PA, USA).

After the instrument with a 40 mm flat plate geometry (TA Instruments, Serial no: 109373; New Castle, DE, USA) was calibrated, the experiment was conducted to check the developed SL gel’s viscosity. The stage temperature was set at 25 °C, temperature equilibration was performed for 120 s, temperature soak time was set for 60 s, and sample test duration was 180 s as per the developed protocol. Samples were loaded based on the size and surface area.

Samples were loaded using an oral dispensing syringe (BD, Lot no.: 9211423; Franklin Lake, NJ, USA). The instrument was then set to “Trim Gap,” and any excess sample was removed using a trimming tool. This was performed to ensure that the sample did not leak outside the periphery of the geometry. Stress vs shear rate data were collected under steady shear conditions by using TRIOS software (Version: 5.0.0.44608) provided by TA Instruments and Microsoft Excel to determine the gel’s viscosity in the units of centipoise (cP).

#### 2.7.3. Liquid Chromatography-Mass Spectroscopy (LC-MS)

An LC-MS analytical method was developed for the quantification of BUP [5,8]. Briefly, chemical evaluation of standards and samples was performed using a 1260 Agilent Infinity II liquid chromatography apparatus (Agilent, Santa Clara, CA, USA) and 6545 XT LC/QTOF mass spectrometer (Agilent, Santa Clara, CA, USA). The mass spectrometry method was established in the scanning mass range of 200–600 *m*/*z*; all the scans were performed under positive ion mode and electron spray ionization (ESI).

**Figure 1:**
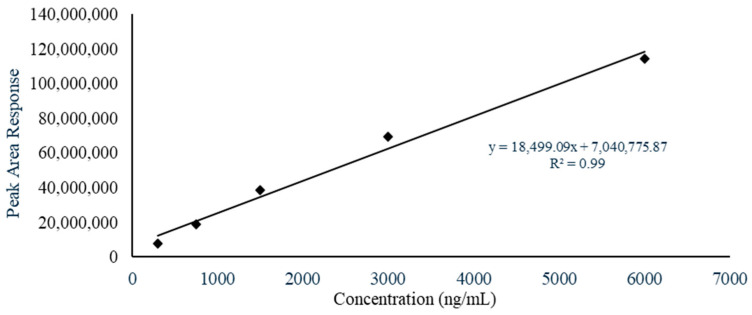
Standard Curve of Buprenorphine.

Calibration of the LC-MS method was performed by establishing a standard curve of BUP. The standard working solutions were subjected to LC-MS analysis using the established analytical method in the concentration range of 300 ng/mL to 6000 ng/mL (Figure 1).

After all the standard working solutions were analyzed, the developed analytical method was utilized during the 30-day study to determine the stability of BUP in the formulated ethanol-free SL gels, stored under the two different storage conditions (*n* = 6). The concentrations of BUP were determined by averaging the concentration values of both sets of SL gels at each storage condition, at each point of the study.

### 2.8. Drug Release Kinetic Studies

SL gels of BUP were prepared based on the protocols mentioned above to achieve the desired concentration of the formulation (75 µg/mL). From the devised SL gels of BUP, 1 mL was transferred to a 10 mL volumetric flask and diluted to volume with water for irrigation (7.5 µg/mL). The solution was then sonicated for 5 min and allowed to reach room temperature. One milliliter of this solution was filtered using a Pall 0.45 µm GHP Acrodisc 13 filter (Waters, Lot no.: 21813904; Milford, MA, USA) and subjected to LC-MS analysis. The drug release kinetics were evaluated at each sampling point by calculating the percentage of drug released from the developed formulation over 90 min.

### 2.9. Functional Group Analysis via FT-IR

FT-IR Spectrometer NicoletTM IsTM 10 (Perkin Elmer, Shelton, CT, USA) with OMNIC software (Version: 9.2.41) was used to determine the chemical interactions of the formulation. A background run was performed before analyzing each sample (62 scans each). BUP, along with other excipients, was analyzed individually as well as in combination using control and BUP-loaded gels. The FT-IR spectra were recorded in the range of 650–4000 cm^−1^, where the runs were carried out once. The presence and interaction between the drug, polymer, and surfactants were checked on day 30. All the signals were compared to identify interactions of BUP with the polymer and each surfactant within the gel matrix.

## 3. Results

### 3.1. Formulation Development of Placebo Gels

Carbomer-based placebo gels appeared to have some turbidity, and the average pH (*n* = 3) was determined to be 4.28 ± 0.04. The viscosity of the gel was within the desired range (*n* = 3) with an average value of 5531.69 ± 891.41 cP. This gelling agent was not chosen for further development due to its acidic nature.

In the case of HEC 250 H, a high-grade polymer was used to formulate the desired gels, and the appearance was clear. The pH was observed to be close to physiological conditions (*n* = 3) with a value of 7.07 ± 0.02. The developed gels were evaluated for their rheological characteristics, and the viscosity was not within the target range (*n* = 3) with an average value of 41,961.00 ± 748.30 cP. It was determined that the viscosity of the gel using this polymer was higher than the desired range. Hence, this polymer was not a suitable candidate for further development.

After the HEC 250 H results were not ideal for further investigation, HEC 250 M, a medium-grade polymer, was subjected to pH and rheological studies. The pH of the HEC 250 M gels (*n* = 3) had an average value of 7.04 ± 0.04, which was like that of the HEC 250 H formulation. For the rheological studies, the average viscosity of the gels devised (*n* = 3) was determined to be 10,346.70 ± 1091.52 cP. The formulated gel’s viscosity was not in the desired range. Thus, this polymer was determined not to be a candidate for further development.

HPMC showed consistent pH values close to physiological conditions, with an average pH of 7.22 ± 0.07. The rheological data showed that the average viscosity of the gels (*n* = 3) was determined to be 3377.01 ± 77.32 cP, and the results were within the desired range. Based on the findings, HPMC was determined to be the ideal candidate for the SL gel formulation with BUP.

### 3.2. Standard Curve of BUP

The developed LC-MS method, as previously described in the literature [5], was utilized for this study. A standard curve was generated by evaluating standard samples of BUP (*n* = 3) within the defined concentration range, demonstrating good linearity (Figure 1). The average coefficient of determination (r^2^) was 0.99, and the recovery values of the standard samples were between 80–120%.

### 3.3. BUP SL Gel Stability Study

A 30-day stability study of the ethanol-free BUP SL gel formulation was performed by assessing the pH change, viscosity, and drug concentration over the course of the study period under two different storage conditions (2–6 °C and 25 °C, 60% relative humidity).

#### 3.3.1. pH Stability of SL Gels During Storage

**Figure 2:**
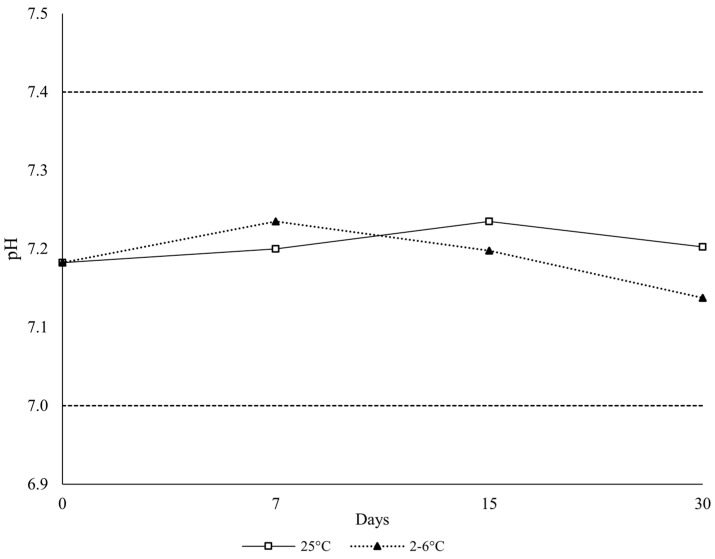
pH Results of the Ethanol-Free Buprenorphine Sublingual Gel Stability Samples Over 30 days.

Assessment of pH change at different sampling points was carried out in triplicate (*n* = 3). The pH of the gels at room temperature was between 7.18–7.24, with an average pH of 7.21 ± 0.02 at 30 days of storage. Refrigerated samples were between 7.13–7.24 with an average pH of 7.19 ± 0.04 at day 30 (Figure 2).

#### 3.3.2. Rheological Stability of Gels

**Figure 3:**
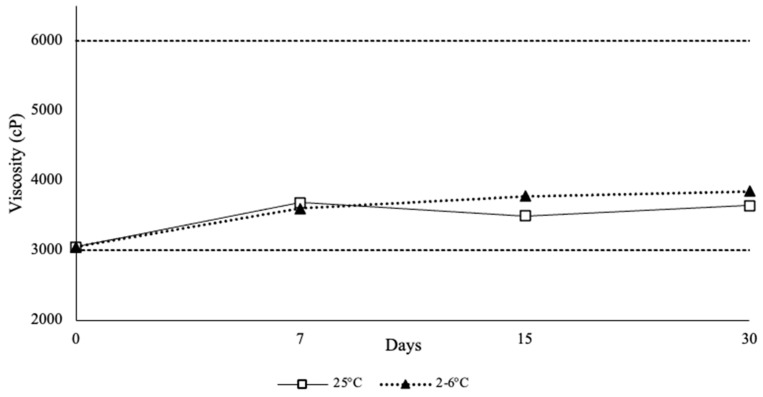
Rheological Results of the Ethanol-Free Buprenorphine Sublingual Gel Stability Samples Over 30 days.

Rheological characterization of the newly formulated SL gels was performed to assess any changes in viscosity over the 30-day study period (*n* = 3) at each storage condition. The results from the stability study were consistent, as the viscosity of the gels was within the range of 3000–3800 cP for both temperature samples, with an average viscosity of 3463.21 ± 288.31 cP for room temperature samples at day 30. For refrigerated samples, the average viscosity was 3564.74 ± 359.02 cP at 30 days of storage (Figure 3).

#### 3.3.3. BUP Content Analysis

**Figure 4:**
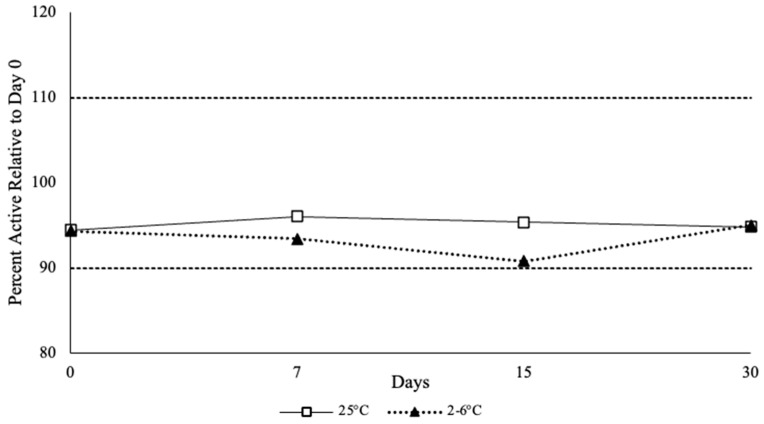
Liquid Chromatography-Mass Spectrometry Results of the Ethanol-Free Buprenorphine Sublingual Gel Stability Samples Over 30 days.

All the formulated ethanol-free SL gels contained BUP between the 90–110% threshold range (94.43–96.01%) for room temperature and (90.79–94.96%) for refrigerated samples, at all-time points. The average concentration relative to the initial concentration was 95.16% ± 0.69 at day 30 for room temperature samples and 93.36% ± 1.83 for refrigerated samples, respectively (Figure 4).

### 3.4. Release Kinetics Study

**Figure 5:**
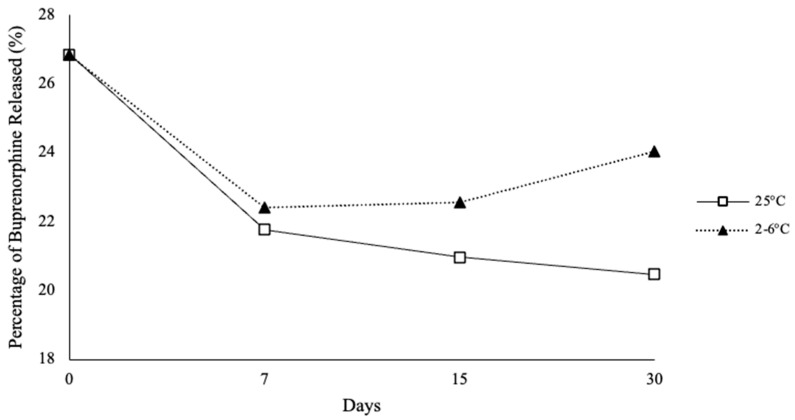
Release Kinetics of Buprenorphine from Sublingual Gel Stability Samples Over 30 days.

Release studies were performed using the developed LC-MS method to ascertain the percentage of BUP released from the devised SL gels (*n* = 3). 22.52% ± 2.9 of BUP was released from the formulation for room temperature samples by 30 days. Refrigerated samples exhibited a release of BUP by day 30 of 23.97% ± 2.1 (Figure 5).

### 3.5. Optimization of HPMC-BUP SL Gels

#### 3.5.1. Penetration Enhancers

Chemical compounds that facilitate the penetration of molecules across poorly permeable biological membranes (oral mucosa) are called penetration enhancers. They are also known as chemical penetration enhancers (CPEs), absorption enhancers, or sorption promoters. CPEs act by increasing lipid bilayer membrane fluidity, intracellular signaling mechanisms, and direct disruption of cell adhesion recognition sequences [9,10].

#### 3.5.2. Choice of Penetration Enhancers

Chitosan and its derivatives enhance drug permeation through mechanisms such as modifying the stratum corneum by acting on tight junctions, disrupting intercellular lipids, and increasing water content in the stratum corneum. However, their use in neonates may pose challenges, including potential toxicity that could lead to embryotoxic effects and long-term adverse impacts on embryo development. Limited water solubility and its absorption interference at neutral and basic pH levels are considered significant drawbacks. Chitosan’s strong binding to the mucosal layer can interfere with drug absorption and reduce drug diffusion. The poor aqueous solubility limits its biomedical utility, particularly in physiological conditions, making it a poor absorption promoter [11,12].

On the other hand, “surfactants,” also known as surface-active agents, are responsible for reducing the surface tension by adsorption. Surfactants are categorized based on the charge of their hydrophilic head. Non-ionic surfactants are recognized for their favorable safety profile, particularly the uncharged nature they possess, which prevents strong electrostatic interactions with cellular membranes and proteins, which can lead to irritation or cytotoxicity often associated with ionic surfactants. This neutral charge minimizes adverse reactions during drug administration, promoting neonatal safety.

#### 3.5.3. Polysorbates

Polysorbates (PSs), such as PS-80 and PS-20, are non-ionic surfactants commonly used in biotherapeutic products. Polysorbates enhance permeation by self-assembling into micelles in aqueous solutions at critical micelle concentrations (CMC). This influences membrane fluidity and drug transport. The amphiphilic nature is crucial for their effectiveness for SL drug delivery [13].

**Figure 6:**
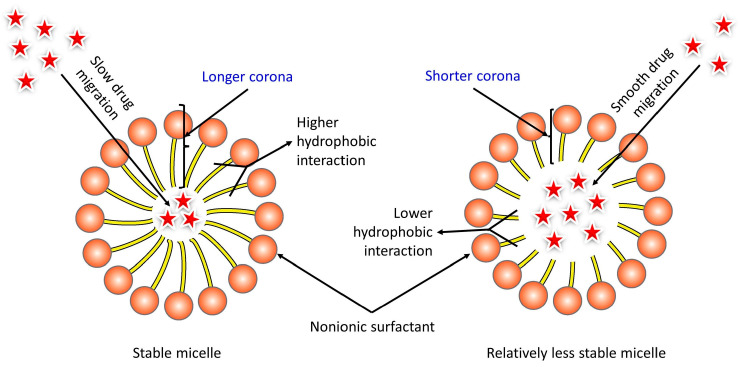
Structure of Micelle Formation and Drug Molecular Interaction: Stable micelle (Poly-sorbate 80) and relatively less stable micelle (Polysorbate 20), and their effect on the drug permeation kinetics and micelle stability.

In an aqueous solution, polysorbate molecules orient themselves with their hydrophilic regions facing water molecules and their hydrophobic regions facing away, reducing the interfacial tension between immiscible liquids. These aggregates form micelles, where the hydrophobic regions face inward, creating a stable structure capable of solubilizing hydrophobic substances in aqueous solutions (Figure 6).

Polysorbate 80, with its ethylene oxide and long hydrocarbon chain (long corona), exhibits both lipophilic and hydrophilic properties, allowing it to partition between lipophilic and hydrophilic domains [14]. Tween 20, with a hydrophilic polyethylene glycol chain (short corona), is highly water-soluble and contributes to its effectiveness in solubilizing hydrophobic drugs in aqueous solutions [15]. The polysorbates offer effective biodegradability, lower toxicity, improved surface and interfacial activity, along with better safety and biocompatibility compared to synthetic alternatives at low concentrations [12]. Hence, these surfactants were considered to be the best choice for the study.

### 3.6. Physicochemical Characteristics of Polysorbate-Containing SL Gels

#### 3.6.1. pH

**Figure 7:**
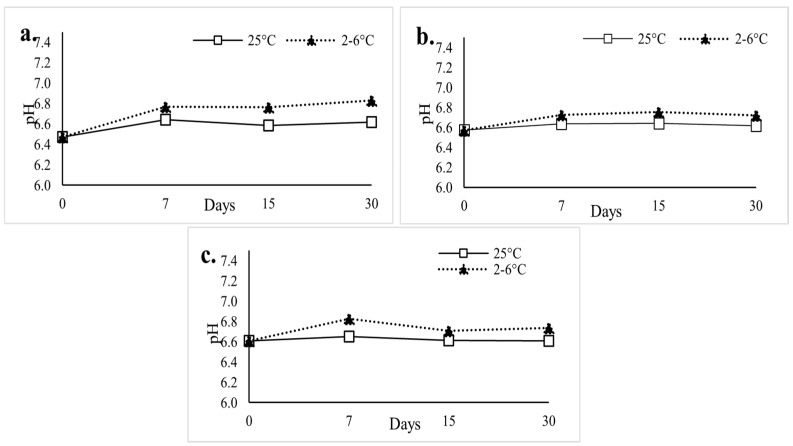
pH Results of ((**a**). Control; (**b**). Tween 20; (**c**). Tween 80 containing) Buprenorphine Sublingual Gel; Stability Samples Over 30 Days.

The pH of Tween-containing BUP SL gels remained consistent over the 30-day stability period (*n* = 3), with values ranging from 6.5 to 6.8. The results, as seen in Figure 7, demonstrated that the incorporation of Tweens did not significantly alter the pH of the gel matrix and was maintained close to physiological conditions. These findings suggest compatibility of the improved SL gel formulation with the oral mucosa and stability at either storage condition.

#### 3.6.2. Rheology

Rheological characterization of the Tween-containing SL gels was performed over the 30-day study period (*n* = 3), at each storage condition. The gel’s viscosity on days 0, 7, 15, and 30 was measured and determined to be within the desired range of 3000–6000 cP for gels stored at both temperature conditions.

**Figure 8:**
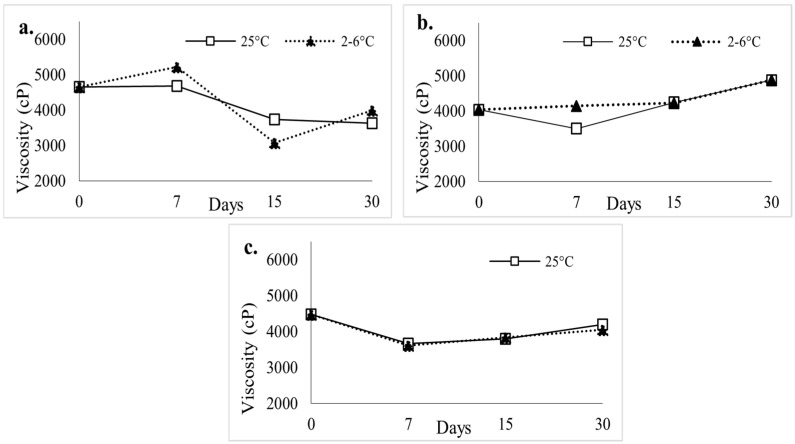
Rheological Results of Tween ((**a**). control; (**b**). Tween 20; (**c**). Tween 80) containing Buprenorphine Sublingual Gel; Stability Samples Over 30 days.

Control gels showed an average viscosity of 4174.74 cP and 4237.08 cP for room temperature and refrigerated conditions, respectively (Figure 8a). Tween 20-containing gels (Figure 8b) had an average viscosity of 4035.17 cP at room temperature and 3992.73 cP for refrigerated samples on day 30. For Tween 80-containing gels, room temperature samples exhibited an average viscosity of 4164.46 cP, and for refrigerated samples, the viscosity was determined to be 4319.63 cP at 30 days of storage (Figure 8c).

#### 3.6.3. BUP Analysis

**Figure 9:**
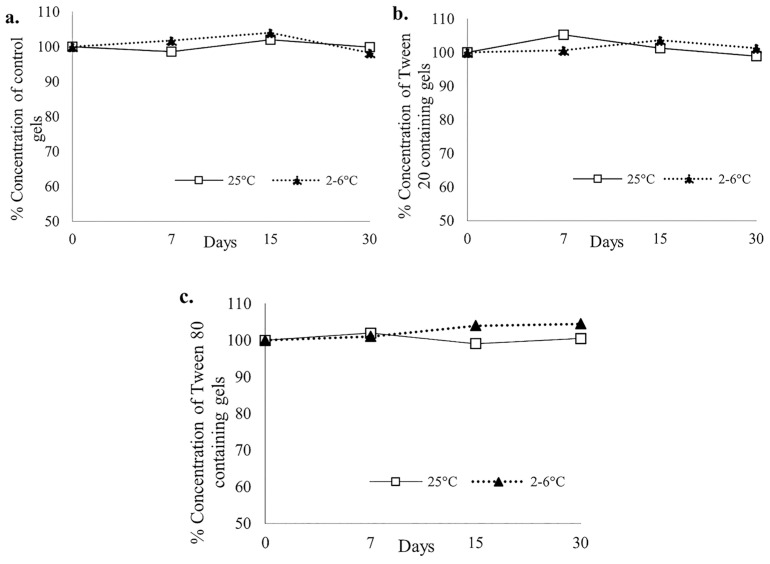
LC–MS results of the ((**a**). control; (**b**). Tween 20; (**c**). Tween 80) containing Buprenorphine Sublingual Gel Stability Samples Over 30 days.

All the formulated ethanol-free SL gels at all the time points contained BUP within the 90–110% threshold range. The controlled samples were between 98.54–102.06% for room temperature and 98.31–104.01% for refrigerated samples. Tween 80-containing gels were between 98.88–105.26% and 100.0–103.6% for room temperature and refrigerated samples, respectively. Tween 20-containing gels were between 99.08–101.95% for room temperature and 100–104.48% for refrigerated samples (Figure 9a–c).

### 3.7. BUP Release Kinetics

**Figure 10:**
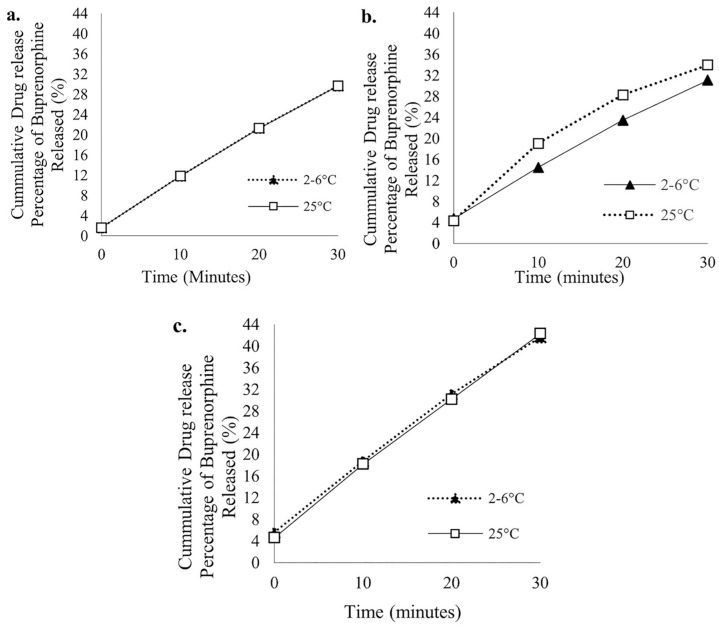
Cumulative drug release results of ((**a**). control; (**b**). Tween 20; (**c**). Tween 80) containing Buprenorphine Sublingual Gel over 30 min.

The drug release studies showed a distinct initial burst release. Among the formulations, Tween 80-containing SL gels (Figure 10c) demonstrated the highest cumulative drug release of 42.3% stored at room temperature and 41.65% for SL gels under refrigerated conditions within 30 min, respectively. On the other hand, Tween 20-containing SL gels demonstrated a drug release of 31.11% and 34% for refrigerated and room temperature samples, respectively (Figure 10b). The control formulation revealed a 29% release of BUP over the studied period (Figure 10a).

The release behavior is indicative of first-order kinetics, suggesting a concentration-dependent release mechanism. The increased drug release from the studied formulations with surfactants, especially Tween 80, was attributed to enhanced solubilization, micelle formation, and improved drug absorption across the gel matrix.

### 3.8. T-Test

To confirm the significance of these findings, statistical analysis was conducted using an unpaired *t*-test to compare the drug release profiles between the developed formulations. The *t*-test confirmed that the variations in drug release between formulations (a vs. b, a vs. c, b vs. c) were significant, having *p*-values less than 0.05. This suggests that the differences in surfactant type and concentration led to meaningful and measurable effects on the drug release kinetics. The data validated that the choice of excipient was critical in enhancing the performance of SL gels and was not due to random variation.

### 3.9. Functional Group Analysis

**Figure 11:**
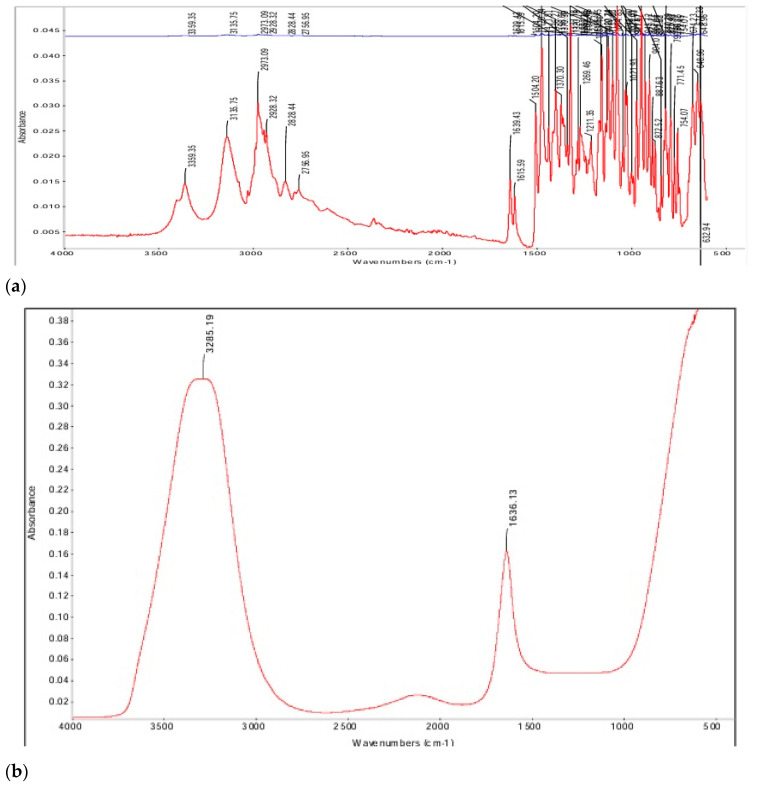

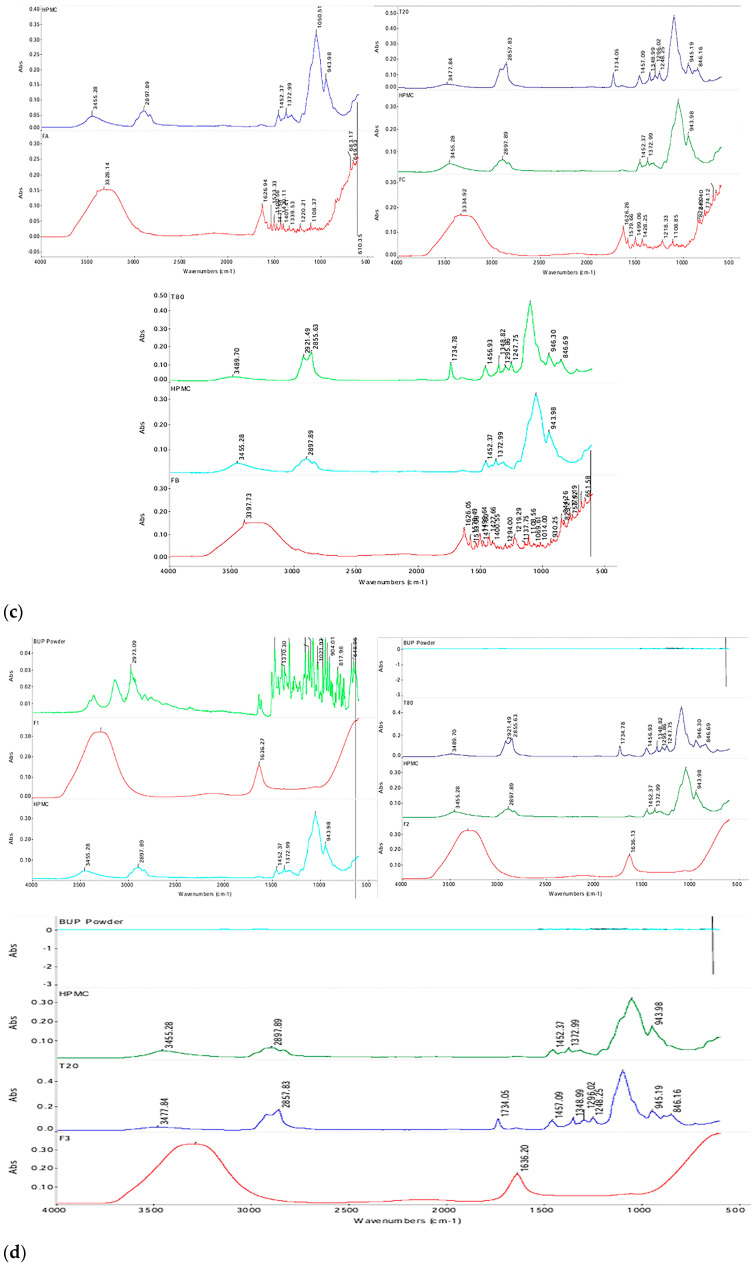
Functional group analysis of the Ethanol-Free Buprenorphine Sublingual Gel Formulation ((**a**). control; (**b**). Tween 80; (**c**). control gels without buprenorphine; (**d**). drug-loaded gels with and without surfactants).

FT-IR analyzes molecular vibrations in a sample by assessing its absorption or transmission of infrared light. FT-IR analysis of the BUP hydrochloride reference standard was determined to have a distinctive peak at 1636.13 cm^−1^, a covalent bond interaction. The reference standard in solution exhibited peaks at 1636.13 cm^−1^ and 3285.19 cm^−1^ (hydrogen bond interaction), respectively. These findings confirmed that BUP was stable in the developed formulation. Additionally, the stacked images of the control and drug-loaded gels indicated no observed chemical interactions (Figure 11).

Upon FT-IR analysis, an HPMC peak was observed at 3450 cm^−1^ in all samples, indicating the presence of a covalent bond interaction. The drug-loaded gels, as well as the polymer with and without surfactants in the gel matrix, were compared (Figure 11d). BUP within the HPMC gel showed a peak at 1636.27 cm^−1^; with Tween 80, a peak was observed at 1636.13 cm^−1,^ and Tween 20 demonstrated a peak at 1636.20 cm^−1^. Peaks for Tween 80 and Tween 20 were not distinctly visible since the amount of surfactants added in the formulation was minute (0.1% *w/v*).

Lastly, individual excipients were measured and recorded. A distinctive characteristic between the Tweens can be understood using their unsaturation peaks. Tween 80 oleic acid moieties had a strong peak at 725 cm^−1^, indicative of =C-H bending. In the case of Tween 20, this peak was absent, since the molecule is a saturated fatty acid. Additionally, a strong peak at 1735 cm^−1^ was observed for both surfactants, indicating C=O stretching. The control gels were evaluated, and the results from the FT-IR analysis indicated that the formulation was compatible with successful drug–polymer interaction (Figure 11c). There was no observed chemical interaction with the addition of surfactants to the formulation.

## 4. Discussion

In our earlier work, our group demonstrated that ethanol-free BUP oral syringes (75 µg/mL) remained physicochemically stable for 60 days and were suitable for treating NOWS. Building on that work, the current study aimed to develop and optimize an ethanol-free SL gel formulation using non-toxic, biodegradable excipients that would not introduce pharmacological activity of their own, which was essential in the consideration for neonatal use. The approach we have undertaken also broadens the potential application of such excipients for other pediatric drug products.

During the initial polymer screening, HPMC (2% *w*/*v*) emerged as the most suitable gelling agent. Unlike the other polymers evaluated, HPMC produced gels with both a physiologically appropriate pH (7.0–7.4) and a viscosity within the optimal range for SL administration (3000–6000 cP). These characteristics supported its selection as the base polymer for further formulation development.

A 30-day stability study confirmed that ethanol-free HPMC gels maintained consistent pH and viscosity under both refrigerated and room temperature storage conditions. These attributes indicated physical stability and suggested that the formulation would be comfortable upon administration to the neonatal oral mucosa. Chemical stability, upon LC-MS analysis, revealed that BUP remained within 90–110% of the labeled concentration of the dosage form throughout the study with minimal observed degradation, further supporting the suitability of the formulation for clinical use. However, only 20–27% of BUP was released from the gel, and these low values can be attributed to the complex gelling structure of the polymer and likely entrapment within the polymer network, which can be an inherent limitation of certain mucoadhesive matrices.

To address this limitation, non-ionic polysorbate surfactants (Tween 20 and Tween 80) were incorporated at 0.1 *w*/*v* to enhance drug solubilization and mucosal penetration. Polysorbate 20 (Tween 20) and polysorbate 80 (Tween 80) were selected for their penetration-enhancing properties and well-established safety profiles in pediatric and neonatal pharmaceutical formulations. These non-ionic surfactants are widely used as solubilizing and stabilizing agents in oral, parenteral, and mucosal drug products due to their favorable biocompatibility and low irritancy potential. Their neutral charge minimizes disruptive electrostatic interactions with biological membranes, making them preferable to ionic surfactants for neonatal applications [14].

The concentration employed in this study, 0.1% *w*/*v*, is lower than or comparable to levels reported in approved pharmaceutical products, where polysorbate concentrations may reach up to 1.0% *w*/*v* depending on formulation type and route of administration [14,15]. Adverse effects associated with polysorbates have primarily been reported in the context of high systemic exposure or prolonged intravenous administration, conditions that differ substantially from the localized, small-volume SL administration intended here.

Polysorbates enhance drug release through micellar solubilization and modulation of membrane fluidity without compromising mucosal integrity [13,14,15]. When used at low concentrations, they align with pediatric formulation principles emphasizing excipient minimization and safety, particularly in vulnerable populations such as neonates [12]. Collectively available literature supports the use of Tween 20 and Tween 80 at 0.1% *w*/*v* as a low-risk and rational strategy to improve BUP release while maintaining an acceptable neonatal safety profile.

These excipients were selected based on their favorable safety profiles, low irritancy potential, and well-established role as penetration enhancers. As expected, inclusion of Tween surfactants substantially increased BUP release, with Tween 80 producing the greatest enhancement. This improvement was consistent with its longer hydrophobic chain, which stabilizes micelles and increases their capacity to solubilize hydrophobic frug molecules. The improved release profiles reflect increased micellar solubilization and altered microstructure within the polymer matrix, which together facilitate more efficient drug diffusion.

Stability assessments of the optimized formulations demonstrated that the addition of polysorbates did not disrupt the physicochemical integrity of the gels. pH remained close to physiological values, and viscosity continued to fall within the desired range, preserving mucoadhesive characteristics and ease of administration. Drug content also remained consistent within acceptable limits over 30 days. FT-IR analysis further confirmed compatibility among BUP, HPMC, and polysorbates, with no evidence of unwanted chemical interactions.

Drug-release studies revealed a clear initial burst release followed by a plateau, consistent with diffusion-controlled kinetics. Importantly, Tween 80-containing gels provided the highest cumulative release—over 40% within 30 min, demonstrating meaningful improvement compared to the control formulation. This enhanced early release is clinically desirable for NOWS, where rapid symptom control is critical. Statistical analyses verified that the observed differences in release between formulations were significant, underscoring the importance of excipient selection in optimizing neonatal SL drug delivery.

Overall, the findings of this study demonstrate that an ethanol-free HPMC-based SL gel containing polysorbate surfactants provides a stable, mucoadhesive platform capable of improving BUP release without compromising safety or physical stability. This formulation addresses key clinical concerns associated with ethanol exposure in neonates and represents a notable advancement in SL drug-delivery strategies for this vulnerable population.

## 5. Conclusions

An ethanol-free BUP SL gel was successfully developed and optimized using HPMC and polysorbate excipients. The formulation demonstrated favorable stability, enhanced drug release, and compatibility with neonatal oral mucosa, representing a promising alternative to ethanol-containing preparations. Further clinical evaluation is warranted to translate these findings into improved clinical care for NOWS.

## Data Availability

The original contributions presented in this study are included in the article. Further inquiries can be directed to the corresponding author.

## Abbreviations

The following abbreviations are used in this manuscript:

BUP	Buprenorphine
LC-MS	Liquid Chromatography–Mass Spectrometry
NOWS	Neonatal Opioid Withdrawal Syndrome
SL	Sublingual
USP	United States Pharmacopeia
